# Exploring the anti-aging potential of phytoestrogens: focus on molecular mechanisms and menopausal symptom modulation

**DOI:** 10.3389/fnut.2025.1651367

**Published:** 2025-09-25

**Authors:** Yuchen Li, Feng Huang, Xin Qian, Chengdong Liu, Yi Yao, Zhangyu Wang, Xiaoyi Zhu, Qi Guo

**Affiliations:** ^1^School of Medicine, Jiangsu University, Zhenjiang, China; ^2^Department of Traditional Chinese Medicine, Affiliated Hospital of Jiangsu University, Zhenjiang, China

**Keywords:** phytoestrogens, menopause management, estrogen receptors, plant-derived therapeutics, clinical translation

## Abstract

The decline in estrogen levels among menopausal women can trigger multisystem dysfunction, significantly increasing the risk of osteoporosis, cardiovascular disease, cognitive impairment, and genitourinary syndrome (GSM). As plant-derived weak estrogen analogs, phytoestrogens demonstrate menopause-related protective potential in preclinical studies by selectively activating ERα, ERβ, and GPER receptor targets to modulate downstream signaling pathways. Current clinical trials indicate their value as an alternative strategy to menopausal hormone therapy (MHT, previously referred to as HRT) for managing menopause-related disorders. Although heterogeneity exists across study outcomes, specific formulations have shown clear efficacy. Future well-designed, large-scale studies are warranted to validate their clinical translational potential.

## Introduction

The menopausal transition, driven by progressive ovarian follicular depletion and declining sex hormone production, represents a critical phase in female reproductive aging (1). Characteristic symptoms—including hot flushes, night sweats, mood disturbances, vaginal dryness, and diminished quality of sex life—primarily arise from hormonal fluctuations during perimenopause, culminating in menopause, defined as 12 months of amenorrhea (2, 3). Beyond these, the decline in estrogen and progesterone levels may disrupt multiple physiological systems—affecting metabolism, cognition, the urogenital tract, and skeletal integrity—thereby increasing the long-term risk of age-related symptoms such as cardiovascular disease, osteoporosis, and cognitive impairment.

Estrogen plays a crucial role not only in regulating female reproductive function but also in neuroprotection, metabolic homeostasis, vascular regulation, and immune modulation through its widely distributed receptors (4). Consequently, estrogen deficiency has been associated with progressive, menopause-related issues in some women. Menopausal hormone therapy (MHT) is a primary intervention for managing menopausal symptoms, though its risk profile necessitates careful consideration. Early Women's Health Initiative (WHI) findings associated oral combined estrogen–progestogen with elevated risks of breast cancer and thromboembolism, but these results were primarily observed in older women (mean age 63) initiating therapy >10 years post-menopause (5, 6). Specifically, the ESTHER trial demonstrated transdermal estrogen's lower venous thrombosis risk compared to oral formulations (7), while the ELITE study revealed reduced atherosclerosis progression only when MHT initiation occurred within 6 years post-menopause (8). Nurses' Health Study data further indicate substantially increased breast cancer risk with prolonged use exceeding 10 years, contrasting sharply with the minimal risk observed in short-term therapy (9). MHT may carry adverse risks in some populations, and given the conflicting evidence, alternative treatments could offer safer options.

Phytoestrogens represent a diverse group of naturally derived non-steroidal plant compounds. Characterized by multiple hydroxyl-substituted aromatic rings, these molecules share a structural resemblance to endogenous estrogens, allowing them to interact with estrogen receptors and elicit estrogen-like or antagonistic biological responses (10). The major categories of phytoestrogens include isoflavones, lignans, coumestans, and resveratrol-like compounds (11). Isoflavones are predominantly found in leguminous plants, such as soybeans, chickpeas, and red clover. Lignans are abundant in flaxseeds, sesame seeds, whole grains, and certain vegetables. Coumestans are rich in sprouted plants, while resveratrol is primarily derived from grapes, peanuts, and related sources (12).

The route of administration of phytoestrogens, as a potential alternative therapy for managing menopausal symptoms, directly influences their bioavailability, efficacy, and safety. Oral administration via tablets, capsules, or beverages is the most common route. However, it is associated with significant first-pass metabolism, a slower onset of action, complex dose–response relationships, and potential antagonistic effects on estrogen receptors at high doses (13, 14). Vaginal local administration for specific symptoms offers advantages such as precise targeting, lower systemic absorption, and a faster onset of action. Nevertheless, it is largely ineffective for systemic symptoms such as hot flushes (15).

Phytoestrogens, primarily isoflavones from soy and red clover, are predominantly marketed in most countries as over-the-counter (OTC) dietary supplements or functional foods. In the United States, they are regulated as dietary supplements; the FDA does not require pre-market approval (16). In the European Union, they are generally managed under the food supplement pathway, and the European Food Safety Authority (EFSA) has conducted safety assessments (17). Canada regulates them as Natural Health Products (NHPs), requiring product licensing and mandatory label warnings (18). Under Japan's Foods for Specified Health Uses (FOSHU) framework, a daily upper intake limit of 30 mg/day for soy isoflavones and specific labeling standards are established (19). Authoritative clinical guidelines do not recommend isoflavones for the treatment of vasomotor symptoms. To date, no prescription drug containing phytoestrogens as the active ingredient has been approved for menopausal symptoms through the medicinal product pathways of either the U.S. Food and Drug Administration (FDA) or the European Medicines Agency (EMA) (20).

In addition, preclinical studies suggest that phytoestrogens may exhibit antioxidant, anti-inflammatory, and antiviral properties. However, clinical evidence remains limited. They are being investigated as potential adjunctive approaches for alleviating menopausal symptoms, reducing osteoporosis risk, and modulating outcomes in hormone-related cancers (21). Given the discordance with current clinical guidelines, this review critically examines the mechanistic basis for phytoestrogens' purported anti-aging effects, evaluates their theoretical potential in managing menopausal conditions, and analyzes translational barriers to evidence-based clinical implementation.

## Molecular mechanisms related to phytoestrogen receptors

Following typical oral intake, phytoestrogens primarily exert their biological effects by binding to specific receptors. This article summarizes the mechanisms of action and signaling pathways of phytoestrogens, with a focus on ERα/ERβ (estrogen receptor alpha/beta) nuclear receptors and the G protein-coupled estrogen receptor (GPER) membrane receptor as the principal targets. Table 1 provides a concise summary of these representative findings.

**Table 1:** Representative phytoestrogens, their receptor targets, signaling pathways, and reported biological effects.

**Phytoestrogen**	**Primary target**	**Signaling pathway**	**Tissue/Model**	**Reported effect**	**Ref**.
Genistein	ERβ	MAPK/PI3K	OVX rats (bone)	↑ bone formation, ↓ inflammation	(29)
Curcumin	ERβ	NF-κB inhibition	SH-SY5Y (AD model)	↓ BACE1, ↓ Aβ production	(30)
S-equol	ERβ, GPER	ERK1/2	Osteoblasts, neurons	↑ ERβ protein, ↑ bone signaling, neurite outgrowth	(32, 40)
Quercetin	ERα	MAPK/ERK	Neurons (Aβ model)	Anti-apoptotic	(33)
Puerarin	GPER	Ca^2^^+^–SIRT1–AMPK	HepG2 cells, obese mice	↓ lipogenesis, ↑ lipolysis	(37)
Kaempferol	GPER	PI3K/AKT/Nrf2	Endothelial cells	↓ oxidative stress, inflammation	(39)
Icaritin	GPER	ERK–NF-κB	Ischemic rats	↓ microglial M1, ↑ M2 (neuroprotection)	(42)

### Nuclear estrogen receptors ERα and ERβ

Phytoestrogens competitively bind to estrogen receptors ERα and ERβ against endogenous estrogens, mediating their effects via classical signaling pathways. Many exhibit higher affinity for ERβ, and this ERβ selectivity contributes to their tissue-specific actions (22, 23). Owing to their predominant dose-dependent relative binding and transactivation preference for ERβ over ERα (24), phytoestrogens may elicit tissue-specific biological responses, including estrogenic-like actions in ERβ-enriched tissues such as bone and vasculature, and modulation of ERα-mediated proliferative signaling in organs, including the breast (25). This receptor selectivity has been proposed to contribute to a more favorable safety profile relative to synthetic estrogens; however, robust clinical evidence confirming reductions in estrogen-sensitive pathologies remains limited. Their efficacy in achieving acute therapeutic endpoints—particularly vasomotor symptom relief—generally appears lower than that of high-potency synthetic estrogen analogs such as ethinyl estradiol (26).

ERβ is expressed at higher levels than ERα in certain tissues, including bone, the cardiovascular system, and specific regions of the central nervous system. This comparison refers only to relative receptor abundance within these tissues and does not imply the diminished functional importance of ERα. In experimental models, ERβ activation can promote apoptosis via mechanisms such as caspase-3 activation, p38/MAPK phosphorylation, and PARP cleavage, suggesting a potential role in maintaining tissue homeostasis and suppressing tumorigenesis (27). However, ERβ signaling exhibits context-dependent effects, and under certain physiological or pathological conditions, its activation may contribute to adverse outcomes (28). In ovariectomized (OVX) osteoporotic rats—a model of postmenopausal bone loss—genistein promotes bone formation and suppresses inflammation via ERβ-mediated MAPK/PI3K signaling cascades (29). In Aβ-treated SH-SY5Y neuronal models of Alzheimer's disease, curcumin demonstrates neuroprotective effects in experimental models by modulating the NF-κB pathway via ERβ, reducing BACE1 expression and Aβ production. Despite curcumin's significant mechanistic promise, its clinical utility is severely limited by low bioavailability and poor absorption, impeding therapeutic translation (30).

Some phytoestrogens may enhance estrogenic signaling in part by upregulating estrogen receptor expression, as shown in selected cellular and animal models. In mouse Neuro-2A neuroblastoma cells, isoflavones increase ERα protein; ERα knockdown reduces isoflavone-induced neurite outgrowth and ER target gene expression, indicating enhanced ERα-mediated signaling (31). Similarly, S-equol upregulates ERβ protein in diabetic osteoporotic rats and osteoblast cells, potentiating bone estrogen signaling (32). In Aβ-induced neuronal apoptosis models, quercetin elevates ERα protein, activating the MAPK/ERK pathway, and inhibiting apoptosis (33). Collectively, data from these models indicate a potential capacity of phytoestrogens to enhance nuclear receptor signaling by upregulating ERα/ERβ protein expression, modulating downstream growth, and survival functions.

### GPER membrane receptor

Distinct from classical nuclear receptor pathways, phytoestrogens can also mediate rapid non-genomic effects through the membrane-bound G protein-coupled estrogen receptor (GPER), activating downstream signaling cascades including cAMP, Ca^2^^+^, MAPK/ERK, and PI3K. Unlike the slower genomic pathways of nuclear ERs, GPER activation typically elicits rapid non-genomic signaling within seconds to minutes, driving transient cellular responses such as ion flux changes or kinase activation. These initial events may subsequently modulate gene expression through secondary messengers (34).

In broiler chickens and primary hepatocytes, genistein promotes glucose uptake via the GPER-cAMP/PKA-AMPK axis, enhancing GLUT2-mediated transport (35). Piceatannol activates GPER-PKA in estrogen-deficient models (OVX mice and 3T3-L1 adipocytes), inducing phosphorylation of hormone-sensitive lipase (HSL) and thereby reducing lipid accumulation (36). Puerarin has been reported to modulate the GPER–Ca^2^^+^–SIRT1–AMPK axis, thereby inhibiting hepatic lipogenesis and enhancing lipolysis, which ameliorates high-fat diet-induced obesity and hepatic steatosis in obese mice and HepG2 cells (37). Regarding cardiovascular protection, 17β-estradiol (E2) predominantly signals through GPER–PI3K to induce vasoprotective effects, whereas phytoestrogens (e.g., daidzein and genistein) exhibit preferential activation of the GPER–PKA pathway to promote vasodilation; however, cross-activation of multiple downstream cascades likely occurs for both E2 and phytoestrogens (38). In ovariectomized mice fed a high-fat diet, kaempferol-induced GPER protein expression upregulation attenuates markers of oxidative stress, inflammation, and apoptosis via PI3K/AKT/Nrf2. This protection—validated in ox-LDL-injured human aortic endothelial cells—is blocked by GPER silencing, confirming target specificity (39).

In the nervous system, S-equol promotes cerebellar neuron migration *in vitro* via GPER-ERK1/2 (40). Genistein activation of GPER/PGC-1α confers neuroprotection in OVX mice with cerebral I/R injury, suppressing NLRP3 inflammasome activation (41). In ischemic rats, icaritin modulates GPER-ERK-NF-κB signaling to suppress harmful microglial activation, shifting them from damaging inflammatory (M1) to protective reparative (M2) states, thereby reducing neuroinflammation (42).

Evidence suggests that GPER can interact with classical ER pathways through context-dependent mechanisms. Its activation induces Src-dependent EGFR phosphorylation, amplifying ERK1/2 signaling, while ERα upregulates EGFR protein levels—creating a feedback loop (43). In certain tumor cells, ERα-dependent estrogenic effects require the concomitant presence of GPER, suggesting a functional interdependence between the two receptors (44). However, direct experimental evidence demonstrating molecular cross-talk between GPER and classical nuclear estrogen receptors (ERα/ERβ) in postmenopausal tissues remains limited and warrants further investigation.

## Phytoestrogen interventions in menopause-associated conditions

Based on the mechanistic insights described earlier, several recent clinical trials have been conducted to assess the anti-aging and symptom-modulating effects of phytoestrogens in postmenopausal women. Table 2 summarizes these studies, which were selected for inclusion in this review.

**Table 2:** Snapshot of recent clinical trials (2019–2025) on phytoestrogens for menopausal symptoms.

**References**	**Study design**	**Phytoestrogen dosage**	**Participants**	**Results**
Barnard et al. (47)	Randomized, 12 weeks	Low-fat vegan diet + 86 g cooked soybeans/day	*n* = 84, postmenopausal women, ≥2 moderate-to-severe hot flushes/day	Intervention significantly reduced hot flushes and improved menopause-related quality of life.
Pokushalov et al. (48)	Randomized, 90 days	25 mg black cohosh +40 mg soy isoflavones + 20 mg SDG lignans/day	*n* = 96, postmenopausal women	Significantly reduced menopausal symptoms, including hot flushes, with a 54.3% improvement in somatic symptoms (*p* < 0.01).
Squadrito et al. (53)	Randomized, 24 months	54 mg genistein/day + calcium + D3	*n* = 200, postmenopausal women with GIO	Both genistein and alendronate had similar effects on BMD, but genistein showed greater effects on certain bone markers.
Lecomte et al. (54)	Randomized, 48 weeks	100 μg of 8-PN/day + calcium + D3	*n* = 100, postmenopausal women with low bone mass	Significantly improved total body BMD in postmenopausal women with osteopenia.
Braxas et al. (60)	Randomized, 12 weeks	108 mg genistein/day	*n* = 54, postmenopausal women with T2DM	Improved FBS, A1C, serum TG, TAC, and MDA in postmenopausal women with T2DM.
Thaung Zaw et al. (61)	Randomized, 24 months	150 mg resveratrol/day	*n* = 125, postmenopausal women	Enhanced cognition, cerebrovascular function, and insulin sensitivity, potentially slowing cognitive decline, especially in late-life women.
Warinsiriruk et al. (65)	Randomized, 12 weeks	6% Pueraria mirifica vaginal gel	*n* = 72, postmenopausal women	Improved vaginal blood flow, VMI, VHI; good safety.
Sritonchai et al. (66)	Randomized, 12 weeks	5% Pueraria mirifica vaginal gel	*n* = 60, postmenopausal women	Improved Nugent score, VHI; no subjective symptom improvement.
Karimi et al. (67)	Randomized, 8 weeks	5% Urtica dioica vaginal cream	*n* = 84, postmenopausal women	Improved vaginal symptoms, ↓pH; good safety.
Bosak et al. (68)	Randomized, 12 weeks	5% chamomile vaginal gel	*n* = 96, postmenopausal women	Improved sexual function comparable to estrogen; favorable safety.
Palma et al. (71)	Prospective, 3 months	75 mg soy isoflavones, BID	*n* = 61, postmenopausal women	Lowering blood pressure, reducing LDL cholesterol.
Yigit and Unsal (74)	Prospective, 6 months	80 mg red clover isoflavones/day	*n* = 75, postmenopausal women with dyslipidemia	Decreased levels of TC, LDL-C, and TG; increased levels of HDL-C.
Kirichenko et al. (76)	Randomized, 2 years	Phytoestrogen preparation (Karinat^®^): 3 capsules/day, each containing 2.5 mg Genistein, 11.8 mg Daidzein, 27.3 mg procyanidin, 4.6 mg flavones, 3.5 mg resveratrol	*n* = 546 (315 early, 231 late postmenopausal)	No IMT effect in early post-menopause; attenuated IMT progression in late post-menopause.
Khapre et al. (77)	Prospective, 12 weeks	40 mg soy isoflavones, BID	*n* = 100, perimenopausal and postmenopausal women	Initiating phytoestrogen supplementation at early menopause may be more beneficial for cardiovascular health.
Schmidt et al. (82)	Randomized, 8 weeks	Rimostil (phytoestrogen complex): 3 capsules/day, containing 7.5 mg Genistein, 35.4 mg Daidzein, 81.9 mg Procyanidin, 13.8 mg Flavones, 10.5 mg Resveratrol	*n* = 66, perimenopausal women with depression	Estradiol improved depression scores; Raloxifene and Rimostil were ineffective; no cognitive differences.

BMD, bone mineral density; 8-PN, 8-prenylnaringenin; FBS, fasting blood sugar; A1C, glycated hemoglobin; TAC, total antioxidant capacity; MDA, malondialdehyde; VMI, vaginal maturation index; VHI, vaginal health index; TC, total cholesterol; LDL-C, low-density lipoprotein cholesterol; TG, triglycerides; HDL-C, high-density lipoprotein cholesterol; IMT, intima-media thickness.

### Hot flushes

Vasomotor symptoms, particularly hot flushes, arise from estrogen deprivation–induced hypersensitivity in hypothalamic thermoregulatory centers—key mediators of temperature homeostasis—coupled with heightened sympathetic activity. MHT is an effective therapy for hot flushes (45), whereas phytoestrogen efficacy remains inconclusive with inconsistent clinical outcomes. Notably, non-hormonal agents such as the neurokinin-3 receptor antagonist fezolinetant have emerged as evidence-based alternatives for vasomotor symptom management (46).

Plant-based diets with soy supplementation reduced hot flush frequency in postmenopausal women, with one study reporting symptom remission in approximately 50% of participants (47). Trials combining black cohosh, soy isoflavones, and SDG lignans also yielded favorable outcomes. Authors attribute the primary effect to black cohosh, which does not directly bind to estrogen receptors, suggesting a mechanism independent of classical estrogen receptor pathways (48).

Current evidence predominantly derives from complex mixtures, with scarce high-quality trials on single phytoestrogen monomers. Future research must prioritize dose–response relationships and molecular mechanisms of individual compounds to enable evidence-based personalized therapy.

### Osteoporosis

The decline in estrogen during menopause accelerates bone resorption and increases osteoporosis risk (49). Phytoestrogens exert anti-osteoporotic effects through receptor-mediated signaling and mitochondrial pathways. For example, glabrene downregulates tartrate-resistant acid phosphatase (TRAP) to inhibit osteoclasts, upregulates osteoprotegerin (OPG) and osteocalcin, and activates Wnt/β-catenin signaling to promote osteogenic genes such as Runt-related transcription factor 2 (Runx2) and Osterix (Osx), restoring bone metabolic balance (50). Beyond classical ER-dependent mechanisms, genistein activates estrogen-related receptor alpha (ERRα), upregulating peroxisome proliferator-activated receptor gamma coactivator 1-alpha (PGC-1α) to promote mitochondrial biogenesis. It also enhances sirtuin 3 (SIRT3) expression, which activates PTEN-induced kinase 1/Parkin (PINK1/Parkin)-dependent mitophagy, reducing reactive oxygen species (ROS), delaying bone marrow mesenchymal stem cell (BMMSC) senescence, and maintaining bone metabolic homeostasis (51).

In a clinical trial conducted in 2018, soy isoflavones were found to reduce bone resorption markers, but their effect on improving bone mineral density (BMD) was minimal (52). However, recent studies have shown that genistein monotherapy may increase lumbar and femoral BMD in glucocorticoid-induced osteoporosis, effects that seem comparable to alendronate (53). Recent studies have also indicated that supplementation with 8-PN standardized hop extract may improve BMD in postmenopausal women with osteopenia, with a positive trend in BMD increase compared to baseline (54).

Overall, clinical evidence suggests phytoestrogens mitigate bone resorption in postmenopausal women, with genistein showing notable efficacy. However, improvements in BMD remain modest, and marked heterogeneity exists among phytoestrogens in both biological activity and clinical outcomes.

### Diabetes mellitus

Estrogen regulates glucose and lipid metabolism by enhancing insulin sensitivity and glucose uptake. After menopause, estrogen decline increases type 2 diabetes in women (55). Estrogen metabolism also accelerates in diabetic women due to hepatic upregulation of SULT1E1 and ABCG2, promoting estrogen inactivation and excretion (56). Phytoestrogens may improve glucose metabolism via estrogen receptor modulation and direct effects on metabolic pathways. Genistein activates PKA and ERK1/2, promoting β-cell proliferation and improving insulin resistance (57). Coumestrol attenuates insulin resistance by modulating sphingolipid metabolism, inhibiting ceramide synthesis, and upregulating SIRT1 to reduce oxidative stress and inflammation (58, 59).

Some studies suggest phytoestrogens improve glucose metabolism in postmenopausal women, but results remain inconsistent. A double-blind trial in diabetic postmenopausal women showed genistein reduced fasting glucose, HbA1c, triglycerides, and improved antioxidant capacity, insulin sensitivity, and HDL cholesterol (60). A 24-month crossover trial reported that low-dose resveratrol reduced fasting insulin and HOMA-IR, suggesting improved insulin sensitivity (61).

Phytoestrogens may improve diabetes-related parameters in postmenopausal women via estrogen-like activity and glucose-lipid metabolic modulation. However, clinical evidence remains heterogeneous, highlighting the need for large, well-designed prospective trials to confirm their therapeutic value.

### Urogenital disorders

Estrogen maintains vaginal elasticity, moisture, and mucosal integrity (62). After menopause, declining estrogen results in epithelial thinning, pH elevation, and disrupted barriers, increasing UTI and GSM risk (63). Estrogen therapy can temporarily alleviate symptoms of vaginal atrophy, and topical phytoestrogen preparations demonstrate unique value.

Experimental evidence suggests phytoestrogens enhance urogenital defense via antimicrobial, anti-inflammatory, and circulatory-promoting actions (64). A systematic review suggests vaginal phytoestrogen formulations outperform oral forms in alleviating vaginal atrophy, incontinence, and sexual dysfunction (15). Clinical trials demonstrate that locally applied phytoestrogen gels/creams**—**including 6% *Pueraria mirifica* gel, 4% soy isoflavone gel, 5% *Urtica dioica* (stinging nettle) cream, and 5% chamomile gel**—**improve vaginal atrophy symptoms, increase Vaginal Health Index scores, enhance epithelial maturation and microvascular perfusion, restore Lactobacillus-dominant microbiota, and improve sexual function (65–68). Notably, 5% chamomile gel demonstrated efficacy comparable to estrogen therapy in improving sexual function (67). In contrast, oral phytoestrogens show minimal efficacy for urogenital symptoms and cannot substitute local therapy (69).

Overall, clinical evidence supports locally applied phytoestrogens for alleviating vaginal atrophy, restoring microbiota, and improving sexual function in postmenopausal women. Despite promising results, standardized formulations and long-term data remain limited.

### Cardiovascular disease

Estrogen protects the cardiovascular system by promoting nitric oxide (NO) generation, regulating lipid profiles, and suppressing inflammation. Menopause-induced estrogen decline increases CVD risks, including atherosclerosis, hypertension, and arrhythmias (70). Phytoestrogens, by binding to estrogen receptors, may mimic these protective effects.

Despite promising *in vitro* and animal results, clinical data remain inconsistent. Studies report mixed outcomes, including lowered blood pressure and LDL (71), improved lipid profiles and endothelial function in meta-analyses, yet also increased carotid intima-media thickness (CIMT), suggesting potential atherosclerosis risk (72).

Research outcomes for isoflavones are conflicting. While some studies suggest that isoflavones have individualized effects on cardiovascular risk factors associated with menopause (73), others report improvements in lipid profiles (74, 75). These discrepancies likely arise from variations in plant source, resulting in isoflavone metabolic profiles and population-specific metabolic heterogeneity.

Cardioprotective effects of phytoestrogens appear time-dependent and vary significantly, influenced by menopausal stage (76), genetics, and comorbidities. The latest research suggests that initiating phytoestrogen supplementation during the early stages of menopause (such as the perimenopausal and early postmenopausal periods) may be more beneficial for cardiovascular health (77). Large, long-term trials are needed to confirm their safety and mechanisms.

### Cognitive disorders

Phytoestrogens exert neuroprotective effects against cognitive impairment through multiple mechanisms, including: activation of the ERβ-mediated PI3K/Akt pathway to promote neuronal survival and synaptic plasticity (78); modulation of the PI3K/Akt-Nrf2 axis to enhance antioxidant capacity, reducing oxidative damage and apoptosis (79); and regulation of the AMPK-PGC1α pathway to improve mitochondrial biogenesis and maintain energy metabolism homeostasis (80). Notably, emerging preclinical evidence suggests that even suboptimal dietary phytoestrogen exposure—in gonadally intact models (adult male mice)—may compromise hippocampal plasticity and remote memory consolidation (81). While this novel perspective underscores the necessity of maintaining adequate levels, its translational implications for postmenopausal cognition require careful interpretation due to fundamental differences in the hormonal milieu between intact males and estrogen-deficient states.

Clinical evidence for the cognitive benefits of phytoestrogens is limited. Resveratrol enhanced cognition, perfusion, and metabolic status over 24 months in older women (61). A randomized trial for perimenopausal depression (PMD) found that phytoestrogens (Rimostil), transdermal estradiol, and raloxifene failed to demonstrate statistically significant improvements vs. placebo. These findings collectively indicate minimal clinical efficacy of estrogenic therapies in PMD management (82). Despite methodological rigor, these negative outcomes—potentially influenced by the short 8-week duration—highlight the need to explore alternative interventions for this complex neuroendocrine condition.

Moreover, specific monomers such as genistein and daidzein show promise as selective estrogen receptor modulators (SERMs) in cognitive trials, particularly for AD (83). S-equol's blood–brain barrier (BBB) permeability further supports its potential, though comparative clinical data remain sparse (84).

Selected studies met the following criteria: (1) primary focus on postmenopausal/peri-menopausal women; (2) intervention with defined phytoestrogen compounds or standardized phytoestrogen-rich extracts; (3) reporting outcomes relevant to menopausal health (vasomotor, bone, metabolic, urogenital, cognitive, and quality of life); and (4) clinical trial design (RCT or prospective cohort with ≥8 weeks duration) published between 2019 and 2025. The excluded criteria included: animal studies, reviews, non-primary data, studies on premenopausal women, or interventions where phytoestrogen effects could not be isolated.

## Bioavailability challenges and interindividual variability

The absorption and metabolism of phytoestrogens in the human body are influenced by multiple factors, with gut microbiota playing a central role. Phytoestrogen precursors predominantly exist in glycosidic forms, and their hydrolysis into aglycones by gut microbiota is essential for systemic absorption (85). Furthermore, gut microbiota biotransform specific phytoestrogens—including isoflavones, lignans, and ellagitannins—into metabolites with greater biological activity and bioavailability, such as equol, enterolignans, and urolithins (86).

Substantial inter-individual variability exists in these processes. A well-documented example is equol production: 50%−70% of Asian populations are equol producers compared with only 20%−30% of Western populations, largely reflecting gut microbiota composition (80). Such ethnic differences contribute to heterogeneity in phytoestrogen bioavailability and clinical outcomes, particularly in interventions involving soy isoflavones. Beyond ethnicity, other determinants—including genetic background, age, sex, health status, and diet—further shape individual differences in absorption and metabolism (86). In addition, the food matrix and the form of intake significantly influence absorption kinetics (87). Compared with purified extracts, phytoestrogens ingested within whole foods display distinct absorption efficiency and plasma concentrations, as food components such as protein and fiber can modulate their release and interact with the gut microbiota (88).

The critical role of bioavailability in determining the physiological impact of phytoestrogens necessitates careful consideration when evaluating their therapeutic potential for menopausal symptoms. While mechanistic studies often highlight the promising biological effects of compounds such as curcumin, its clinical translation is significantly hampered by its inherently low bioavailability (89). Furthermore, heterogeneity in clinical trial outcomes assessing phytoestrogens for menopausal symptom relief can be partly attributed to substantial variations in bioavailability (90). This variation stems from the diverse formulations used (complex mixtures vs. isolated compounds, capsules vs. whole foods) and significant inter-individual differences among participants, including gut microbiota composition, which profoundly influences absorption and metabolic activation (91). Consequently, inconsistent bioavailability complicates the interpretation of efficacy data and hinders robust comparisons between studies. To enhance the reliability and clinical relevance of future research, methodological refinements are essential. These include the standardized use of well-characterized monomeric compounds with defined pharmacokinetic profiles and the strategic stratification of participant populations based on key determinants of bioavailability, such as enterotype or metabolic phenotypes.

## Discussion

Phytoestrogens modulate estrogen signaling through multi-receptor targeting, offering a multi-target strategy for addressing menopause-related dysfunction. In osteoporosis, they bind to ERs, inhibit the RANKL-RANK pathway, suppress osteoclast activity, and activate the Wnt/β-catenin pathway to improve bone density and structure (92). In diabetes, the targets extend to the PPAR and AMPK pathways, enhancing insulin sensitivity, promoting cholesterol transport, and reducing inflammation (93). Different phytoestrogens vary in receptor affinity and activity, leading to diverse effects. Although mechanistic complexity increases, this model provides potential for systemic, multi-pathway regulation.

Given the potential of phytoestrogens to improve menopausal health, further studies on their mechanisms and efficacy are needed. Current clinical evidence on hot flushes and cardiovascular benefits remains inconsistent (94). First, most positive outcomes are based on multi-component formulations or plant-based dietary interventions, whose synergistic effects may surpass those of standardized single-compound preparations. Evidence for the efficacy of phytoestrogen monomers remains limited, with significant heterogeneity among studies (94). Second, substantial individual differences in the metabolism and bioavailability of phytoestrogens exist, heavily influenced by gut microbiota composition. The proportion of S-equol producers is considerably higher among East Asian women compared to Western populations, which may partially explain inter-population differences in clinical outcomes (95). Third, factors such as chemical structure, dosage form, and food interactions can affect oral absorption rates, resulting in variable plasma concentrations and treatment effects (96). Fourth, different phytoestrogens possess varying affinities and activation capacities for ER subtypes (ERα, ERβ), and some effects are dose-dependent, yet the optimal therapeutic window remains undefined. In addition, population heterogeneity—including menopausal stage, baseline comorbidities, and genetic background—may significantly influence intervention outcomes and should be carefully addressed in future study designs.

Phytoestrogens hold multi-target value in managing menopausal symptoms. Local formulations with puerarin, soy isoflavones, and nettle show good safety and efficacy (15). Genistein displays effects comparable to alendronate in GIOP via ERβ/ERRα and mitochondrial pathways (53). S-equol and resveratrol show promise in cognitive protection by improving cerebral blood flow and reducing β-amyloid (61). Composite plant-based interventions also effectively alleviate vasomotor symptoms, with strong clinical feasibility (47, 97).

Phytoestrogens offer a natural multi-target option in menopausal care. Challenges include heterogeneity, low bioavailability, and individual variability. Future research should focus on standardized monomer studies and precision interventions to optimize safety and efficacy as MHT alternatives.
